# Utilizing high-density mapping for ablation of mitral annular flutter in a patient with persistent left superior vena cava

**DOI:** 10.1016/j.hrcr.2023.12.006

**Published:** 2023-12-13

**Authors:** Alex Cubberley, Hafiza Khan, Mustafa Dohadwala

**Affiliations:** Baylor Scott & White, The Heart Hospital Plano, Plano, Texas

**Keywords:** Atrial fibrillation, Mapping, Perimitral flutter, Ablation, Persistent left superior vena cava

**Key Teaching Points**

• Mitral annular flutter should be investigated as a cause of recurrent atrial arrhythmias in patients with known persistent left superior vena cava.
• High-density activation mapping can be used to link to structures when activations are absent or missing.
• Using local activation time history to find isochronal points where the fewest points are present may suggest the critical isthmus as point of ablation to terminate mitral annular flutter.

## Introduction

Persistent left superior vena cava (PLSVC) is a thoracic congenital vascular anomaly that occurs owing to failure of regression of the superior cardinal vein that rarely drains into the coronary sinus (CS).^1^ It is commonly associated with other congenital anomalies such as single ventricles, tetralogy of Fallot, and atrioventricular septal defects but can present as an isolated or as an incidental finding.^2^ PLSVC is suspected to be an arrhythmogenic source of atrial flutter and fibrillation owing to convergence of tissues derived from separate embryonic mesenchymal cells developing into separate types of myocardial tissue.^3^^,^^4^ We present a case of using high-density mapping to detect and ablate mitral annular flutter in a patient with PLSVC and prior ablation.

## Case report

Our patient is a 65-year-old man with a history of mechanical aortic valve replacement for a bicuspid aortic valve and persistent atrial fibrillation. Two years prior to presentation, the patient had undergone initial ablation with pulmonary vein and posterior wall isolation, with acute entrance and exit block (30–40 W, 20 mL/min, SmartTouch, Surround Flow; Biosense Webster, Irvine, CA). He developed recurrent episodes of symptomatic atrial fibrillation with elevated heart rates in 2022. Electrocardiogram demonstrated atrial fibrillation, 86 beats per minute, with QRS 112 ms, QTC 425 ms, and with incomplete right bundle branch block.

The patient was taken to the electrophysiology lab for redo ablation of his atrial fibrillation. Preprocedural computed tomography with 3D reconstruction (TeraRecon) demonstrated persistent left-sided superior vena cava. The veins were largely isolated aside from an area along the superior left upper pulmonary vein (LUPV) antrum. There was also a gap along the prior roof line. There was rotational activity along the left atrial septum and LUPV antrum (Cartofinder; Biosense Webster). The LUPV antrum, the gap along the roof, and the septal rotational activity were ablated (40 W, 20 mL/min, SmartTouch, Surround Flow; Biosense Webster). The patient went into atrial flutter with cycle length of 270 ms with eccentric coronary sinus activation. Entrainment of anterior left atrium (LA) and distal CS confirmed mitral annular flutter. Our conventional plan would have been to perform ethanol injection of the vein of Marshall (VOM) followed by endocardial mitral annular flutter ablation and, if needed, CS ablation. Owing to PLSVC, the initial ablation set entailed a conventional inferolateral endocardial mitral line.

**Figure 1:**
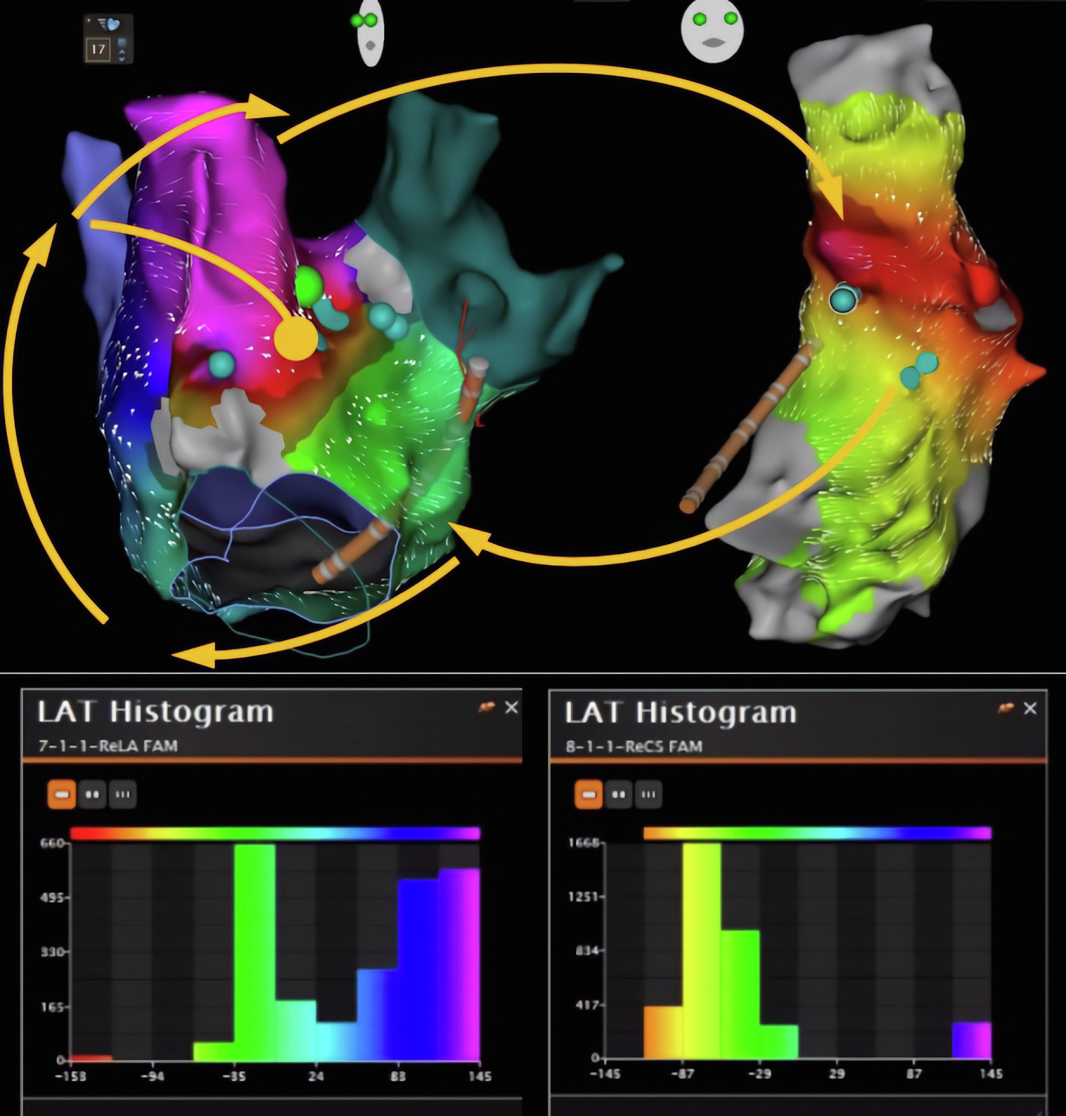
Between -60 ms and +145 ms, tachycardia was active within the left atrium. The tachycardia then traveled to the persistent left superior vena cava (PLSVC) through a small channel, evidenced by small number of isochronal points in the PLSVC–coronary sinus (CS)–great cardiac vein map at +145. The tachycardia then traveled from PLSVC to CS and entered the left atrium. LAT = local activation time.

The tachycardia persisted. Following this, ablation within the distal CS, great cardiac vein (GCV) ostium, and PLSVC was performed in proximity to the endocardial lesion set (30 W, 20 mL/min, SmartTouch, Surround Flow; Biosense Webster). Only areas where the PLSVC was in contact with the appendage, coumadin ridge, and LUPV were ablated; ablation of the free wall of the PLSVC was not performed to avoid perforation. Despite extensive ablation, tachycardia did not terminate, and it was unclear if the connection between the LA and PLSVC was broad or discreet. Using an 8-spline, 3mm-3mm-3mm spaced catheter (Octaray; Biosense Webster), dense activation and voltage mapping was performed of the LA as 1 structure and the PLSVC, CS, and GCV as a separate combined structure. The distal CS was used as timing reference for the activation mapping (Figure 1).

**Figure 2:**
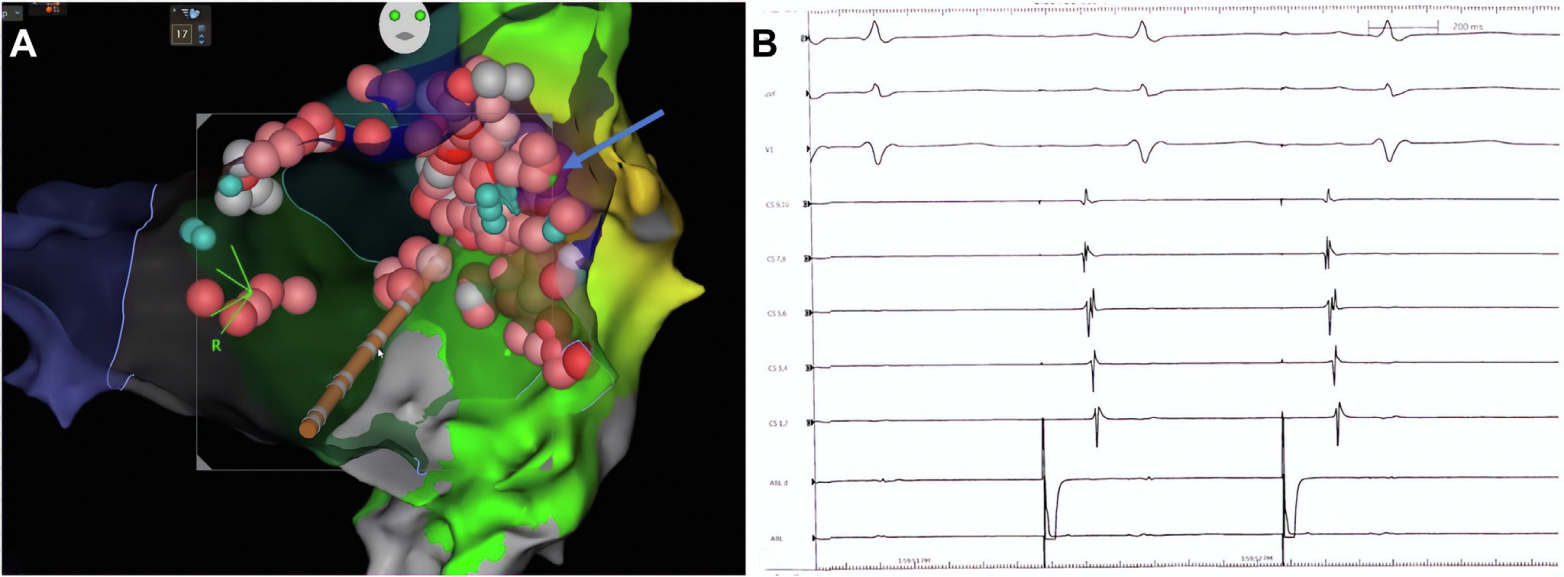
**A:** Anteroposterior view of the left atrium post radiofrequency ablation of left atrial roof, right pulmonary vein antrum, and perimitral circuit with ablation points on medial aspect of persistent left superior vena cava (PLSVC)–coronary sinus (CS) junction. Green tag indicates point of termination of tachycardia (*arrow*). Teal tags indicate areas of heavy fractionation. **B:** Electrogram demonstrating proximal-to-distal CS activation when pacing from left atrial appendage, confirming mitral annular block after ablation of the PLSVC-CS junction.

At timing point +145 ms, a large portion of the left atrial appendage base was active, evidenced by activation map and local activation time (LAT) histogram. In the PLSVC-CS-GCV map, only a small isochronal area at +145 ms was present, evidenced by LAT histogram, suggesting this to be narrow isthmus connecting the LA and the PLSVC (Figure 1, Supplemental Video 1). Although targeted lesions in this location within the PLSVC were unsuccessful (see teal ablation tags, points of heavy fractionation), ablating just adjacent to this area in the LA led to tachycardia termination (green ablation tag, arrow) and block across the mitral isthmus (Figure 2). Ablation points were titrated to an ablation index of 350–400 at 30 W within the PLSVC-CS-GCV while targeted to an ablation index of 450–500 at 40 W within the LA. Power was not titrated during ablation points of the PLSVC. The patient was discharged home the same day on metoprolol succinate and warfarin without an antiarrhythmic. He remained in sinus rhythm when seen in follow-up 3 months later.

## Discussion

We present a case of successful mitral annular flutter ablation in a patient with PLSVC. In patients with PLSVC, a conventional approach to radiofrequency catheter ablation can be challenging owing to the altered anatomy of the coronary sinus system and associated structural cardiac anomalies. Moreover, ethanol injection is not possible for the PLSVC to facilitate block. VOM ablation could not be performed, as the VOM was not present in our case given the PLSVC. Normally, the VOM lies within a vestigial fold of pericardium known as the ligament of Marshall, which itself is an atretic remnant of the embryonic left subclavian vein that lies within the mitral isthmus. Balloon occlusion and ethanol injection of the VOM allows for ablation of the posterior mitral isthmus.^5^ Balloon occlusion and ethanol injection of the PLSVC would only occlude blood return from the left upper extremity and would likely only dilute ethanol systemically, having no ablative antiarrhythmic effects on the left atrial tissue.

There has been 1 other reported case of ablation of perimitral annular flutter with PLSVC but without the use of high-density activation mapping.^6^ Another study has shown successful 1-year outcomes with isolation of the PLSVC in patients with atrial fibrillation.^5^ However, in that study, mitral annular block was not attempted. In our case, we demonstrate the value of high-density voltage and activation mapping to attain a successful mitral annular block with PLSVC. High-density mapping provides detailed anatomic mapping to avoid ablation of the free wall of the PLSVC, which may provide safety from perforation. Also, novel tools such as LAT histogram may lead to successful outcomes when otherwise they may have failed. In this case, targeting the earliest and smallest isochronal area on LAT histogram helped achieve conduction block in a tachycardia traveling from one compartment (ie, LA) into another compartment (ie, CS-PLSVC-GCV).

## Conclusion

High-density activation mapping is a powerful tool to guide mitral annular flutter ablation in PLSVC. It can guide safer ablation within the CS-PLSVC-GCV and efficiently locate fibers connecting the 2 “separate compartments.”

## Appendix Supplementary Data

Supplemental Video 1 Activation mapping of demonstrating propagation of perimitral flutter through isthmus connecting LA and PLSVC.
